# Supplementary material for: Parkinson’s Disease Through the Lens of Metabolomics: A Targeted Systematic Review on Human Studies (2019–2024)
Source: J Clin Med. 2025 Sep 5;14(17):6277. doi: 10.3390/jcm14176277 (PMC12429492; doi:10.3390/jcm14176277)
Supplement: Supplementary file 1 [file jcm-14-06277-s001.zip › jcm-3770499-supplementary.pdf]

SUPPLEMENTARY MATERIAL

Parkinson's Disease Through the Lens of Metabolomics: A Targeted Systematic Review on Human Studies (2019–2024)

Federico Cannas[1†], Karolina Krystyna Kopeć[1†], Natalia Zuddas[2], Flaminia Cesare Marincola[2*], Giorgio Arcara[3], Michele Loi[4], Michele Mussap[4,5] and Vassilios Fanos[4]

[1] Department of Mechanical, Chemical and Materials Engineering, University of Cagliari, 09123 Cagliari, Italy; fcannas1@gmail.com (F.C.); k.kopec@studenti.unica.it (K.K.K.)

[2] Department of Chemical and Geological Sciences, University of Cagliari, Monserrato (CA), Italy; flaminia@unica.it (F.C.M.); nataliazuddas02@gmail.com (N.Z.)

[3] IRCCS San Camillo Hospital, 30121 Venice, Italy; giorgio.arcara@hsancamillo.it

[4] Neonatal Intensive Care Unit, Department of Surgical Sciences, University Hospital of Cagliari, University of Cagliari, 09042 Monserrato (CA), Italy; micheleloi1994@gmail.com (M.L.); mmumike153@gmail.com (M.M.); vafanos@tiscali.it (V.F.)

[5] Laboratory Unit, Department of Surgical Sciences, University Hospital of Cagliari, University of Cagliari, Cagliari, Italy

† These authors contributed equally to this study.

* Author to whom correspondence should be addressed: flaminia@unica.it

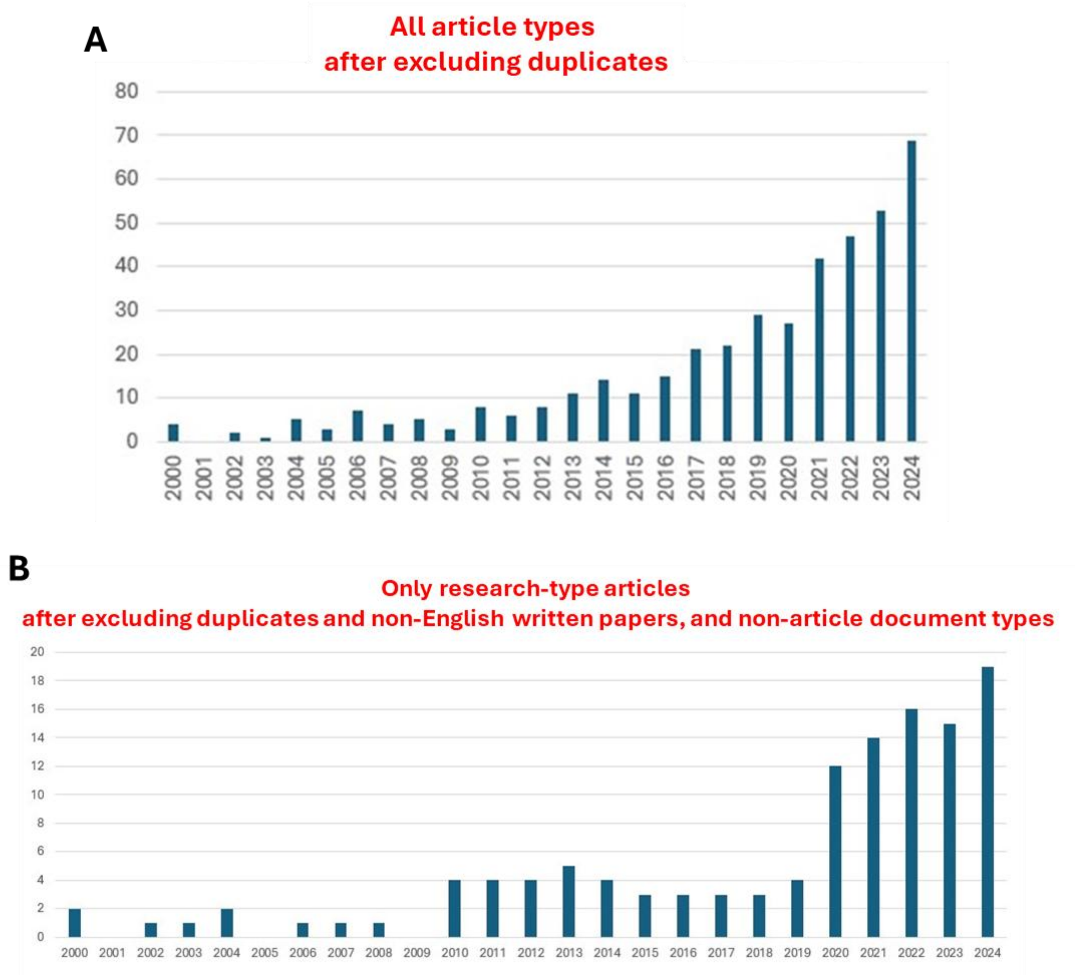

Figure S1. Annual *s*cientific *p*roduction *from 2000 to 2024* based on Bibliometrix *analysis.* A) Distribution of 417 articles screened after duplicate removal. B) Distribution of *120* articles after duplicate removal*,* excluding non-English publications and non-article document types.
